# Evidence of object permanence, short-term spatial memory, causality, understanding of object properties and gravity across five different ungulate species

**DOI:** 10.1038/s41598-024-64396-8

**Published:** 2024-06-14

**Authors:** Alina Schaffer, Anja Widdig, Ruben Holland, Federica Amici

**Affiliations:** 1 Behavioral Ecology Research Group, Institute of Biology, University of Leipzig, Leipzig, Germany; 2 Department of Human Behavior, Ecology and Culture, Max Planck Institute for Evolutionary Anthropology, Leipzig, Germany; 3 Zoo Leipzig, Leipzig, Germany; 4 Research Group Human Biology and Primate Cognition, Institute of Biology, University of Leipzig, Leipzig, Germany; 5 Department of Comparative Cultural Psychology, Max Planck Institute for Evolutionary Anthropology, Leipzig, Germany

**Keywords:** Cognition, Inference, Gravity, Permanence, Memory, Object properties, Ungulates, Psychology, Short-term memory, Spatial memory

## Abstract

In their natural environment, animals face a variety of ecological and social challenges, which might be linked to the emergence of different cognitive skills. To assess inter-specific variation in cognitive skills, we used ungulates as a study model, testing a total of 26 captive individuals across 5 different species (i.e., dwarf goats, *Capra aegagrus hircus*, llamas, *Lama glama*, guanacos, *Lama guanicoe*, zebras, *Equus grevyi*, and rhinos, *Diceros bicornis michaeli*). Across species, we used the same well-established experimental procedures to test individuals’ performance in naïve physics tasks, i.e. object permanence, short-term spatial memory, causality, understanding of object properties, and gravity. Our results revealed that study subjects showed object permanence, were able to remember the position of hidden food after up to 60 s, and inferred the position of hidden food from the sound produced or not produced when shaking containers. Moreover, they showed an understanding of basic object properties, being able to locate objects hidden behind occluders based on their size and inclination, and could reliably follow the trajectory of falling objects across different conditions. Finally, inter-specific differences were limited to the understanding of object properties, and suggest that domesticated species as goats might perform better than non-domesticated ones in tasks requiring these skills. These results provide new information on the cognitive skills of a still understudied taxon and confirm ungulates as a promising taxon for the comparative study of cognitive evolution.

## Introduction

In their natural environment, animals face a variety of ecological and social challenges. Theories of cognitive evolution suggest that these challenges have favoured the emergence of cognitive skills that allow individuals to better cope with the problems they encounter in their ecological niches^1,2^. One of the most basic cognitive skills is object permanence, which is the ability to know that objects continuously exist through space and time, even when they are hidden^3^. In humans, object permanence develops through six main stages: in the first three stages (i.e., stage 1: development of reflexes, stage 2: development of habits, stage 3: development of coordination between vision and prehension), infants show no object permanence^3^. Before approaching the first year of age, however, they understand objects as separate entities existing even when they are not visible (stage 4) and can locate objects after visible (stage 5) and invisible displacements (stage 6)^3–6^. Object permanence is also widespread in species other than humans^7^, for example, it has been shown in primates^8–16^, birds^17–24^, dogs (*Canis familiaris*;^25–27^), cats (*Felis catus*;^27,28^), goats (*C. a. hircus*;^29–31^), sheep (*Ovis orientalis aries*;^31,32^), giraffes (*Giraffa carmelopardalis*;^33^) and dolphins (*Tursiops truncatus*;^34^), just to name a few.

Another crucial cognitive skill is the ability to recall the position of objects or other animals after different delays of time. Short-term spatial memory, for instance, is very common in different taxa^7^, and has been reported in primates^8,12,35^, birds^36–38^, dogs^25,39^, cats^25,27,39^, goats^29^, sheep^40^, giraffes^33^ and horses (*Equus caballus*;^41^) among others. Studies in ungulates, a less studied taxon in this regard, have shown that giraffes can successfully recall the position of hidden food after delays of up to 30 s^33^, whereas horses can store information about hidden food for at least 20 s^41^.

Causal understanding, defined as the understanding that one event is the consequence of another, might also be important to deal with socio-ecological challenges^42^. Causal understanding, for instance, might facilitate the retrieval of embedded food (e.g. trap tube task:^43,44^; e.g., tool-use in crows:^45^) and allow individuals to better predict where and when food will be available^46^. From 3 years of age, human children are able to solve causality tasks in which they are presented with two opaque containers, only one being baited, and they receive either acoustic or visual cues about the location of the reward, by for instance shaking either the baited or the non-baited container^47^. Species other than humans can also use acoustic cues to infer the location of food, including primates^48^, corvids (*Garrulus glandarious*;^49^), pigs (*Sus scrofa domestica*), and wild boars (*Sus scrofa scrofa*;^50^). Pigs and wild boars, for instance, were able to locate food in one out of two containers, if the baited one provided an acoustic cue when being shaken. However, when the empty container was shaken, they failed to infer that the food was in the other container^50^. When using visual cues (i.e., lifting the baited or the non-baited container), also goats and sheep showed an understanding of causal relationships^31^.

The ability to understand object properties is also crucial for several species. In humans, infants from 5 months of age understand object properties like solidity, and at 9 months they preferentially search for objects where a protuberance marks a hidden object under a cloth lying flat on a table, suggesting that these skills may be part of our innate core knowledge^5,6,51–57^,but see^58^. Moreover, when seeing one object disappear behind one out of two occluders of different size and/or shape, children from 3.5 months of age preferentially search for the object behind the occluder having the proper size and/or shape to hide the object^57^. These skills might be very useful also for species other than humans. By understanding which objects can visually occlude others, for instance, animals might make predictions about where predators might be hiding^22,59,60^. Newborn domestic chicks (*Gallus gallus*), indeed, can successfully locate an object behind the only occluder compatible with the object’s shape, and they do it without any previous experience with objects, suggesting that these skills might be part of their innate core knowledge^22^. However, experience might also be important to acquire a better understanding of object properties, as has been shown in horses^61^ and pigs^50^.

Finally, animals can also rely on other object properties, like gravity, to effectively solve socio-ecological challenges. Humans, for instance, show the first evidence of gravity understanding from 7 months of age, looking longer at a test event with inappropriate acceleration where a ball moved up-/downward while speeding up/slowing down^62–65^. However, also other species can use gravity as a cue to locate food in different experimental contexts. Several species, for instance, can successfully locate falling objects, including great apes (*Gorilla gorilla, Pongo pygmaeus, Pan troglodytes, Pan paniscus*;^66^), cotton-top tamarins (*Saguinus oedipus oedipus*;^67^), and dogs^68^. However, when the trajectory of the falling object gets redirected (e.g. by letting objects fall through crossed tubes), children show a gravity bias until around 3 years of age, failing to account for the presence of the tubes and still searching below the releasing point^66,69^. This gravity bias is present in several other species, including cotton-top tamarins^70^, macaques (*Macaca mulatta*, *Macaca arctoides*^71^), and dogs^68^, whereas great apes can successfully locate food falling through crossed tubes^66^.

Although these naïve physical cognitive skills (i.e. object permanence, short-term spatial memory, causality, understanding of object properties, and understanding of gravity) are likely crucial to face a variety of socio-ecological challenges, it is currently unclear how these skills are distributed across species, and which factors best predict this distribution. Some of these skills, for instance, object permanence and understanding of object properties, might be part of the innate core knowledge of several taxa^22^, although experience might also be important to acquire a better understanding of object properties^50,61^. Other skills, however, might have emerged in different species as a response to the specific socio-ecological challenges faced during evolution and might be largely independent of the living conditions experienced by single individuals. Researchers have proposed different evolutionary hypotheses on the distribution of cognitive skills, which are not mutually exclusive. Here, we will focus on the three hypotheses that have been widely explored in other studies.

First, some authors have proposed that species with larger dietary breadth (i.e. consuming a higher number of dietary categories) may more likely exploit novel food sources and might have thus evolved enhanced cognitive skills to better cope with this variation^72^. In primates, there is indeed evidence that larger dietary breadth is linked to enhanced cognitive skills such as inhibition, but it is still unclear whether dietary breadth also has the same explanatory power in other taxa^73^. Second, species with high levels of fission–fusion dynamics (i.e. experiencing frequent changes in subgroup size and composition; high level meaning highly fluid with either relatively stable or flexible subgroup membership;^74^) might show an increase in some cognitive skills, like memory to remember the identity and social relationships of other group members that are often in other subgroups, and inferential skills to effectively deal with fragmentary information about absent group members^74,75^. Third, domesticated species have been selected for skills and traits that facilitate their interaction with humans and are usually considered to be more playful and explorative than their wild counterparts^76,77^. Therefore, domesticated species may be more interested in anthropogenic objects and more likely to explore them, and thus might have a higher chance to acquire important information on their properties during their lives^29,50,78^.

In this study, we aimed to assess how different cognitive skills are distributed across captive individuals belonging to different ungulate species. We selected ungulates as a study model for two main reasons. Firstly, despite being economically crucial for humans, ungulates are a still largely under-studied taxon^30,79,80^. Secondly, ungulate species show an impressive variety of socio-ecological characteristics^81^ and thus constitute an ideal model to contrast different evolutionary hypotheses on the emergence of cognitive skills. In this study, we compared the performance of dwarf goats (*C. a. hircus*), llamas (*L. glama*), guanacos (*Lama guanicoe*), Grevy´s zebras (*E. grevyi*) and rhinos (*D. b. michaeli*) in a series of tasks testing their naïve physics, i.e. object permanence, short-term spatial memory, causality, understanding of object properties and understanding of gravity. We used the same controlled experimental procedures for all study subjects to allow more accurate comparisons^8,31,66,82^.

Based on existing literature (e.g.,^83–90^), we predicted that all species would show object permanence (Prediction 1), as this is part of their core knowledge, but that there would be inter-specific variation in the other tasks. In particular, if dietary breadth explained the distribution of cognitive skills across taxa, we would predict that species consuming a higher number of dietary categories (i.e., goats) would perform better than the others (i.e., llamas, guanacos, Grevy´s zebras and rhinos) in the other tasks (Prediction 2a). If fission–fusion levels explained the distribution of cognitive skills, we would instead predict that species with higher levels of fission–fusion dynamics (i.e., goats, Grevyi’s zebras) would perform better than species with lower levels of fission–fusion dynamics (i.e., llamas, guanacos, rhinos), especially in tasks requiring memory and inferential skills (i.e., the short-term-spatial-memory and the causality tasks, see below; Prediction 2b). If domestication explained the distribution of cognitive skills across taxa, we would predict that domesticated species (i.e., goats, llamas) would perform better than non-domesticated species (i.e., guanacos, Grevy’s zebras, rhinos; Prediction 2c).

## Methods

*Ethics*. Informed consent was obtained from welfare managers at the zoo of Leipzig, who controlled and approved all the procedures. All the animals participated on a completely voluntary basis and no invasive procedures were used. During the task, individuals were never water or food deprived, and motivation to participate was ensured exclusively by the use of highly preferred food belonging to their natural diets. The experiments thus provided a form of enrichment for the subjects and did not present any risks or adverse effect. The study was carried out in accordance with the national regulations of Germany and ARRIVE guidelines. The Office of the Ethics Advisory Board at the University of Leipzig confirmed that formal ethical approval was not required.

*Subjects.* We tested 26 subjects belonging to 5 ungulate species, including 7 dwarf goats (*C. a. hircus*), 3 llamas (*L. glama*), 5 guanacos (*L. guanicoe*), 8 Grevyi zebras (*E. grevyi*) and 3 rhinos (*D. b. michaeli*). For each species, we tested all individuals that were housed in the Leipzig Zoo (Germany) and that interacted with the experimenter and the setup on a voluntary basis. All subjects were housed with conspecifics in enclosures with inner and outer areas and were all individually recognizable thanks to their distinctive morphological features. For more information about the study subjects, see Table 1. All study subjects received a daily diet consisting of hay and were able to eat grass or leaves ad libitum when they were in their outside enclosures. They were also provided daily with pellets, carrots, and, for the rhinos, apples, which keepers brought to the enclosure within plastic buckets and then spread on the ground.

**Table 1:** List of all study subjects, including their species, sex, and the number of trials they run for each task and condition.

Species	Subject	Sex	Number of trials per task and condition												
			Permanence					Properties				Gravity			
			Permanence	Memory	Control	Shake full	Shake empty	4 × 16	16 × 4	flat	Control	Gravity	Control	Vertical tubes	Crossed tubes
Goat	Goat1	Female	12	24	12	12	12	12	12	12	12	–	–	–	–
	Goat2		12	24	12	12	12	12	12	12	12	12	12	12	12
	Goat3		12	24	12	12	12	12	12	12	12	12	12	12	12
	Goat4		12	24	12	12	12	–	–	–	–	12	12	12	12
	Goat5		12	24	12	12	12	12	12	12	12	12	12	12	12
	Goat6		12	24	12	12	12	12	12	12	12	12	12	12	12
	Goat7		–	–	–	–	–	–	–	–	–	12	12	12	12
Llama	Sancho	Male	12	24	12	12	12	12	12	12	12	12	12	12	12
	Krümel		12	24	12	12	12	12	12	12	12	12	12	12	12
	Flax		12	24	12	12	12	12	12	12	12	12	12	12	12
Guanaco	Phebe	Female	12	24	12	12	12	12	12	12	12	12	12	12	12
	Rike		12	24	12	12	12	12	12	12	12	12	12	12	12
	Lissitha		12	24	12	12	12	12	12	12	12	12	12	12	12
	Maike		12	24	12	12	12	12	12	12	12	12	12	12	12
	Lolitha		12	24	12	12	12	12	12	12	12	12	12	12	12
Zebra	Franz	Male	11	24	12	11	9	–	–	–	–	–	–	–	–
	Babule		12	24	12	12	12	12	12	12	12	12	12	12	12
	Mats		12	24	12	12	12	12	12	12	12	12	12	12	12
	Dolly	Female	3	11	4	7	4	12	12	12	12	3	1	1	1
	Nina		12	24	12	12	12	12	12	12	12	12	12	12	12
	Nora		12	24	12	12	12	12	12	12	12	12	12	12	12
	Lilian		12	24	12	12	12	9	5	10	8	1	0	0	1
	Petra		2	7	3	2	1	–	–	–	–	–	–	–	–
Rhino	Saba	Female	12	24	12	12	12	12	12	12	12	12	12	12	12
	Sarafine		12	24	12	12	12	12	12	12	12	12	12	12	12
	Vungu	Male	12	24	12	12	12	12	12	12	12	12	12	12	12

*Experimental procedure.* We conducted three different tasks, whose procedures were largely based on previous studies conducted in other species^9,48,91–94^. Individuals were tested in their social groups, so a trial was only started when the subject was not interacting with other group members and was observing the experimenter. During all trials, the experimenter wore sunglasses to avoid providing subjects with unconscious cues about the location of food. All trials were video-recorded and scored online, and videos were later used to assess inter-observer reliability, which was very good (object permanence k = 0.994, object properties k = 0.936, object gravity k = 1.000). To avoid learning effects, we used up to 12 trials per individual and condition.

**Figure 1:**
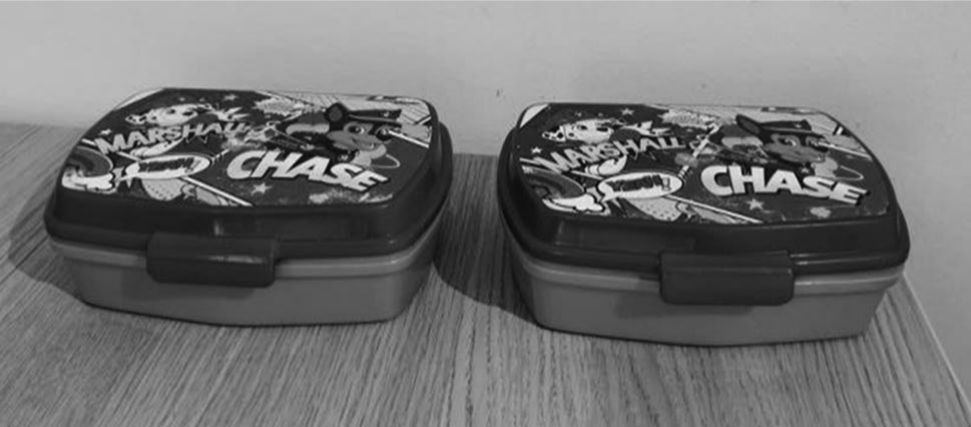
Setup for the object permanence, short-term spatial memory and causality tasks, consisting of two identical containers.

The first task aimed to assess object permanence, short-term spatial memory and causality (see Fig. 1). It consisted of a habituation phase (to habituate subjects to the set-up and procedures) and an experimental phase (which included 6 conditions, for a total of 72 experimental trials per subject). In the habituation phase, we first baited one out-of-reach opaque container in full view of the subject, after 5 s we closed it and pushed it closer to the subject. If within 30 s the subject approached the container (i.e., came with its muzzle or tongue within at least 20 cm from it), the experimenter opened it and let the subject eat the food. After four successful trials (out of five consecutive ones), we repeated this procedure, with the following exceptions: (i) we used two identical containers instead of one; (ii) only one container was baited, in full view of the subject; (iii) both containers remained open, so the subject could choose the container while the food was visible. If the subject approached the baited container within 30 s, it was allowed to eat the food; if the subject approached the empty container, the content of the other container was shown and then the food and containers were removed. After four successful trials (out of five consecutive ones), the subject proceeded to the experimental phase.

**Figure 2:**
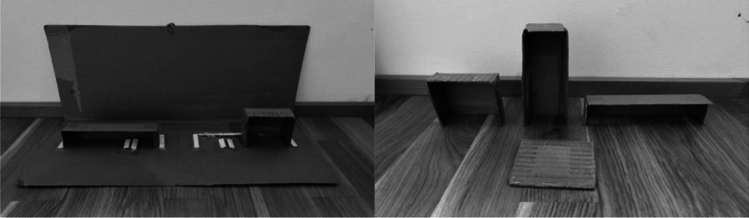
Setup for the object properties task, consisting of a board with attached panels, an occluder (left) and different panels (right).

**Figure 3:**
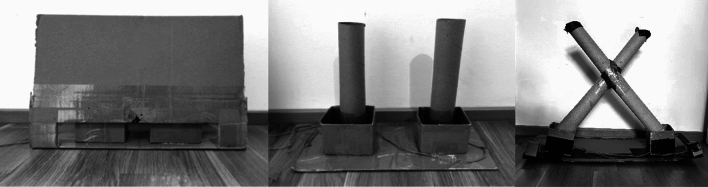
Setup for the gravity task, as seen by the study animals, including the container condition (left), the vertical tubes condition (middle) and the crossed tubes condition (right).

In the experimental phase, we administered all the conditions in a pseudo-randomized order, and counterbalanced the position of food across trials and conditions, without locating food more than three times on the same side. In the *object permanence condition* (12 trials)*,* we tested whether individuals understood that objects continued to exist even when out of view. We used two identical opaque containers and only baited one, as in the second part of the habituation phase. The experimenter simultaneously closed the containers and pushed them closer to the subject, who could make a choice. In the *short-term memory condition*, we tested whether individuals could retain information about the location of food after a delay of 30 or 60 s. We used the same procedure as in the object permanence task, but before pushing the containers closer to the subject the experimenter, we waited either 30 s (12 trials) or 60 s (12 trials), while staring at the floor to provide no inadvertent cues to the subject. In the *shake full condition* (12 trials), we tested individuals’ understanding of causality and, in particular, whether they could use the sound produced by non-visible food to infer its position. We baited one of the two containers out of the subject’s view and shook the baited container three times (thus producing a clear rattling sound) before pushing the containers closer to the subject. No visual cue was provided to the subject as to the position of food. The *shake empty condition* (12 trials) was identical, except that the experimenter shook the empty container instead of the baited one. This condition allowed ruling out that successful subjects in the shake full condition were not simply selecting the shaken cup because of stimulus enhancement. Finally, we included an *olfactory control condition* (12 trials), to control whether individuals could rely on olfactory cues to locate non-visible food. We used the same procedure as in the object permanence condition, except that the container was baited out of the subject’s view, so that the subject had no cues other than olfactory ones to locate the food. For goats, llamas and guanacos, we used pellets, while for zebras and rhinos we used small pieces of carrots (approx. 1 × 2 cm for zebras and 3 × 3 cm for rhinos).The second task aimed to assess individuals’ understanding of object properties, and it consisted of 4 conditions, for a total of 24 experimental trials per subject (see Fig. 2). We administered all conditions in a pseudo-randomized order, and counterbalanced the position of food across trials and conditions, as above. The three *panel conditions—horizontal, vertical,* and *flat* (6 + 6 + 6 trials) tested whether individuals could infer the panel that could occlude a food reward, based on the relative shape and position of the food and the occluder. Food could only fit behind one of the two panels, even if rotated. In the *panel horizontal condition* (6 trials), the experimenter placed two different opaque panels (one squared 8 × 8 cm and one horizontal 4 × 16 cm) perpendicular to the ground, one on the right and one on the left side, and hid them to the subject’s view with a vertical occluder. Then the experimenter showed a piece of food to the subject, above and in the middle of the two panels, holding it with both hands. Both hands were lowered behind the occluder and simultaneously moved behind the two panels, baiting one of the two. Then the experimenter removed the occluder and pushed the panels closer to the subject, which could make a choice. In these three conditions, we used the same procedure, except that the two panels differed from each other in shape or position. We used the following pairs of panels, respectively: (i) one horizontal 4 × 16 cm panel and one 8 × 8 cm panel, both perpendicular to the ground; (ii) one vertical 16 × 4 cm panel and one 8 × 8 cm panel, both perpendicular to the ground; and (iii) one 8 × 8 cm panel, perpendicular to the ground, and one 8 × 8 cm panel, lying flat on the ground. We also added the *panel control condition*, where two identical opaque panels (each 8 × 8 cm) were used, to test whether the experimenter inadvertently provided cues to the subjects during the experimental procedure. We used carrots for all species, adapting their size to the panel size (sticks that were approximately 6 cm long were hidden behind 8 × 8 cm panels and those that were 12 cm long were hidden behind 16 × 4 cm or 4 × 16 cm panels).The third task aimed to assess individuals’ understanding of gravity, and it consisted of 4 conditions, for a total of 48 experimental trials per subject (see Fig. 3). Administered all conditions in a pseudo-randomized order, and counterbalanced the position of food across trials and conditions. In all conditions, the experimenter used two identical opaque containers, showed the subject that they were empty by inclining them towards the individual, and placed them on a board. In the *containers condition* (12 trials), we assessed whether individuals searched for a falling object in the container below the releasing point. In this condition, the experimenter covered the upper part of the containers with an occluder, simultaneously raised both hands (i.e., on top of each container), showed the content of the hands (i.e. one with food, one empty) on the tips of the fingers, and then simultaneously opened the hands to let the food fall into the container below. The experimenter removed the occluder and pushed the board closer to the subject, who could choose one container, as above. In the *vertical tubes condition* (12 trials), we assessed whether individuals searched for an object falling inside a tube, in the container below the releasing point. In this condition, the two containers were connected to two vertical opaque tubes. The procedure was identical to the previous condition, except that no occluder was used and the food fell inside the tubes. In the *crossed tubes condition* (12 trials), we assessed whether individuals showed a gravity bias, and in particular, whether they searched for a falling object below the releasing point, even in the presence of crossed tubes. The procedure was identical to the previous condition, except that we used two crossed tubes instead of two vertical ones. Finally, we included a *gravity control condition* (12 trials)*,* to control whether the experimenter inadvertently provided cues to the subjects (including acoustic cues) during the experimental procedure. The procedure was identical to the container condition, except that the experimenter’s hands were maintained below and behind the occluder during the baiting procedure, thus providing no visual cue to the subject about the position of the food. For all species, we used pieces of carrots (approximately 2 × 2 cm, except for rhinos, where we used 3 × 3 cm pieces).

*Statistical analyses.* We used the package glmmTMB^95^ in R (R Core Team 2020) to run three generalized linear mixed models^96^, one for each of the tasks we conducted (i.e. object permanence, object understanding, gravity). We prepared three different datasets, entering one line for each subject and trial (object permanence: N = 1704; object understanding: N = 1040; gravity: N = 1014). The number of data points in each dataset was not identical, because the number of trials and conditions differed across tasks (see Methods), and because few individuals stopped participating in the tasks before they were over (see Table 1). In all the models, our binomial response was whether the subject made the correct choice (i.e. choosing the baited side) or not. In all models, we included as test predictors the 2-way interaction of species and condition (to assess whether performance differed across species depending on the condition). We further included trial number (*z*-transformed), food position (right or left), and subject’s sex (female or male) as controls, and the subject’s identity as a random factor. We then used likelihood ratio tests (LRT) to compare each of the full models described above to a null model that was identical but excluded test predictors^97^. If the full model significantly differed from the null model, we used the drop1 function to assess whether the interaction included was significant, and in case it was not, we re-ran the model after removing the interaction, while keeping the main terms. In case of significant test predictors, we used the emmeans package to assess estimates for the single levels of the predictors^98^. We checked model assumptions with the “DHARMa” package^99^ and the “performance” package^100^. We detected no problems of convergence, overdispersion, or multicollinearity (maximum variance inflation factors across models = 2.52)^101^.

To assess whether subjects performed above chance (0.50) in the different conditions, we also conducted a Wilcoxon test. To this end, we prepared three further datasets, one for each of the three tasks we conducted, entering one line for each subject, with the average of correct responses in each condition (object permanence task: N = 25; object understanding task: N = 22; gravity task: N = 22). Below, we only report the *p* values of significant (i.e. *p* ≤ 0.05) tests.

## Results

**Figure 4:**
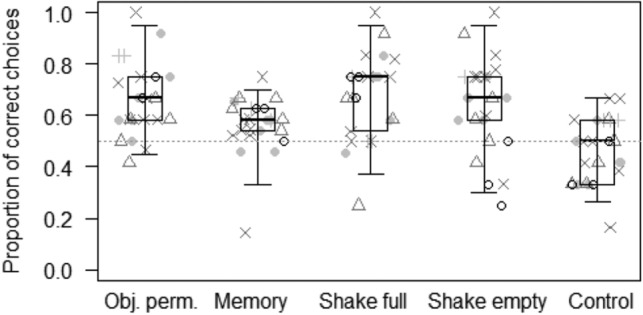
Proportion of correct choices in each condition of the object permanence task. Light grey dots represent average values for each individual goat, light grey pluses for each guanaco, dark grey triangles for each llama, dark grey crosses for each rhino, and black circles for each zebra. Thick lines represent the median of the individual values for each condition, the horizontal ends of the box represent the 25% and 75% quartiles, and the ends of the whiskers represent the 2.5% and 97.5% quartiles. The grey dotted line represents chance level.

In Model 1, we tested whether the probability of making the correct choice in the object permanence task varied depending on the interaction of species and conditions. The full model significantly differed from the null model (GLMM: LRT *χ*^2^ = 60.68, *df* = 24, *p* < 0.001)), with condition (but not species) having a significant effect (Table 2). In particular, the probability of making the correct choice was lowest in the olfactory control condition (mean ± SD: 0.46 ± 0.03), and higher in all the other conditions (object permanence: 0.66 ± 0.03; short-term spatial memory: 0.59 ± 0.02; shake empty: 0.63 ± 0.03; shake full: 0.68 ± 0.03; see Fig. 4). In contrast, species had no significant effect (Table 2). Wilcoxon tests further showed that, except for the control condition, subjects performed above chance levels in all the other conditions (object permanence: *p* < 0.001; short-term spatial memory: *p* < 0.001; shake empty: *p* = 0.002; shake full: *p* < 0.001).

**Table 2:** Results of the three models run, including estimates, standard errors (SE), 95% confidence intervals (CIs), likelihood ratio tests (LRT), degrees of freedom (df), and *p* values for each test predictors (marked with an asterisk when significant) and for each control (in italics), with the reference category in parentheses.

MODELS	Estimate	SE	2.5–97.5% CI	*LRT*	*Df*	*P*
*Model 1: Probability of making the correct choice in the object permanence task*						
Intercept	0.32	0.13	0.06 to 0.57	–	–	–
Species (guanaco)	− 0.06	0.15	− 0.34 to 0.23	2.09	4	0.720
Species (llama)	0.14	0.24	− 0.33 to 0.60			
Species (rhino)	− 0.17	0.18	− 0.51 to 0.18			
Species (zebra)	− 0.02	0.15	− 0.32 to 0.28			
Condition (object permanence)	0.30	0.15	0.00 to 0.59	35.28	4	< 0.001*
Condition (control)	− 0.51	0.15	− 0.80 to − 0.22			
Condition (shake empty)	0.18	0.15	− 0.12 to 0.47			
Condition (shake full)	0.40	0.15	0.10 to 0.70			
*Trial number*	− 0.04	0.05	− 0.14 to 0.05	0.75	1	0.385
*Food position (right)*	− 0.02	0.10	− 0.22 to 0.18	0.04	1	0.838
*Sex (male)*	0.14	0.16	− 0.17 to 0.45	0.78	1	0.376
*Model 2: Probability of making the correct choice in the object property task*						
Intercept	0.18	0.19	− 0.19 to 0.55	–	–	–
Species (guanaco)	− 0.59	0.20	− 0.97 to − 0.20	11.74	4	0.019*
Species (llama)	− 0.09	0.32	− 0.71 to 0.53			
Species (rhino)	− 0.49	0.24	− 0.95 to − 0.03			
Species (zebra)	− 0.50	0.21	− 0.90 to − 0.10			
Condition (4 × 16)	0.08	0.18	− 0.28 to 0.44	15.40	3	0.002*
Condition (control)	− 0.06	0.18	− 0.42 to 0.30			
Condition (flat)	0.60	0.19	0.23 to 0.97			
*Trial number*	− 0.05	0.07	− 0.18 to 0.07	0.68	1	0.408
*Food position (right)*	0.94	0.13	0.68 to 1.20	52.07	1	< 0.001
*Sex (male)*	− 0.04	0.22	− 0.46 to 0.39	0.03	1	0.865
*Model 3: Probability of making the correct choice in the gravity task*						
Intercept	0.10	0.18	− 0.25 to 0.45	–	–	–
Species (guanaco)	0.01	0.18	− 0.34 to 0.37	2.83	4	0.586
Species (llama)	0.12	0.32	− 0.50 to 0.75			
Species (rhino)	0.28	0.23	− 0.17 to 0.73			
Species (zebra)	− 0.09	0.22	− 0.52 to 0.35			
Condition (control)	− 0.67	0.19	− 1.03 to − 0.30	14.59	3	0.002*
Condition (crossed tubes)	− 0.32	0.19	− 0.68 to 0.05			
Condition (vertical tubes)	− 0.14	0.19	− 0.51 to 0.23			
*Trial number*	0.00	0.07	− 0.13 to 0.13	0.00	1	0.974
*Food position (right)*	0.87	0.13	0.61 to 1.13	44.18	1	< 0.001
*Sex (male)*	0.23	0.23	− 0.23 to 0.69	0.96	1	0.327

**Figure 5:**
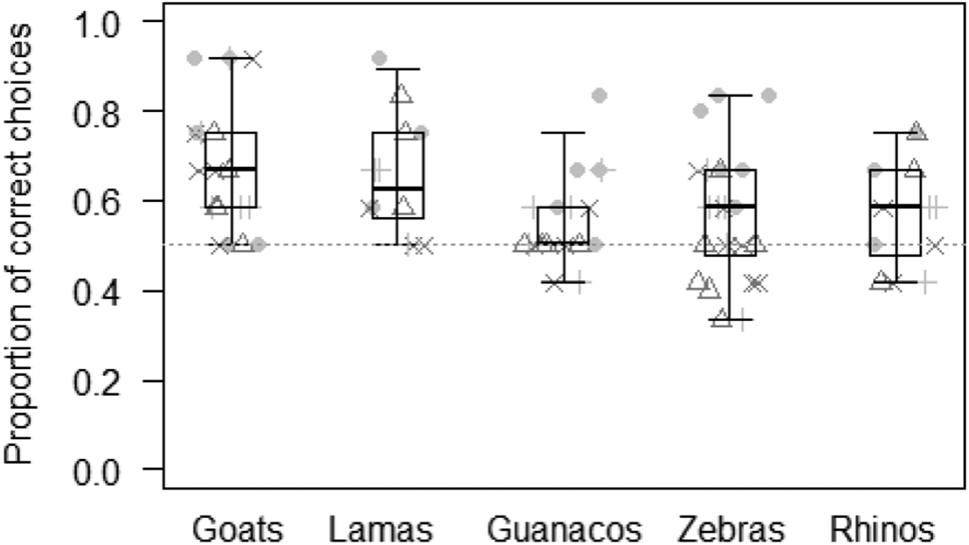
Proportion of correct choices for each species in the object properties task. Light grey dots represent average values for the flat condition, light grey pluses for the 4 × 16 condition, dark grey triangles for the 16 × 4 condition, and dark grey crosses for the control condition. Thick lines represent the median of the individual values for each condition, the horizontal ends of the box represent the 25% and 75% quartiles, and the ends of the whiskers represent the 2.5% and 97.5% quartiles. The grey dotted line represents chance level.

**Figure 6:**
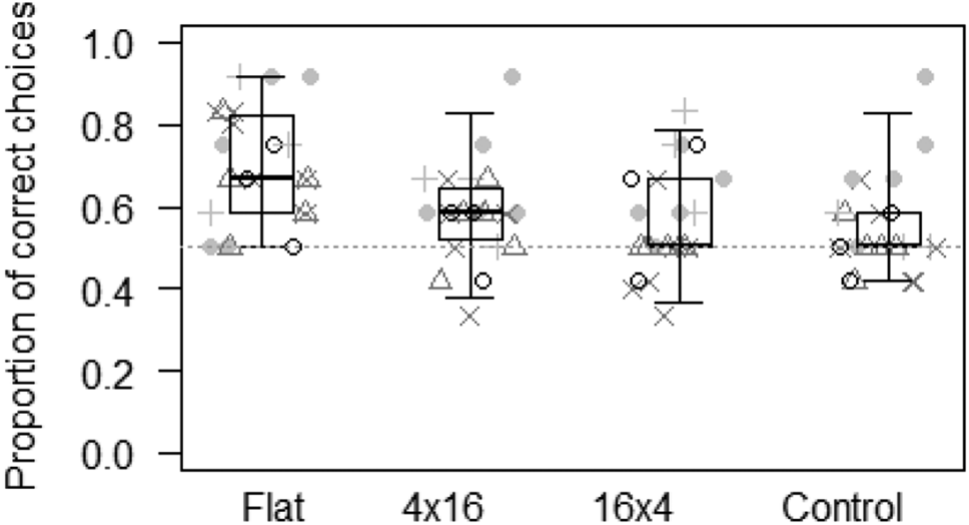
Proportion of correct choices in each condition of the object properties task. Light grey dots represent average values for each individual goat, light grey pluses for each guanaco, dark grey triangles for each llama, dark grey crosses for each rhino, and black circles for each zebra. Thick lines represent the median of the individual values for each condition, the horizontal ends of the box represent the 25% and 75% quartiles, and the ends of the whiskers represent the 2.5% and 97.5% quartiles. The grey dotted line represents chance level.

In Model 2, we assessed whether the probability of making the correct choice in the object property task varied as a function of the interaction between species and condition. The full model differed from the null model (GLMM: LRT *χ*^2^ = 35.55, *df* = 19, *p* = 0.012). Here, both species and condition showed a significant effect on the probability of making the correct choice (Table 2). In particular, goats (0.69 ± 0.04) and llamas (0.67 ± 0.05) performed the best, followed by rhinos (0.57 ± 0.04), zebras (0.57 ± 0.03) and guanacos (0.55 ± 0.04; see Fig. 5). Moreover, the probability of making the correct choice was highest in the flat condition (0.71 ± 0.03), and lower in the others (4 × 16: 0.59 ± 0.03; 16 × 4: 0.57 ± 0.03; panel control: 0.56 ± 0.03; see Fig. 6). Wilcoxon tests showed that, except for the control condition, subjects performed above chance levels in all the other conditions (flat: *p* < 0.001; 4 × 16: *p* = 0.006; 16 × 4: *p* = 0.046).

Finally, in Model 3, we tested whether the probability of making the correct choice in the gravity task varied depending on the interaction of species and conditions. We found a significant difference between the full and the null model (GLMM: LRT *χ*^2^ = 34.37, *df* = 19, *p* = 0.017), with a significant effect of condition (Table 2). In particular, the probability of making the correct choice was lowest in the gravity control condition (0.51 ± 0.03), and higher in the other conditions (crossed tubes; 0.60 ± 0.03; vertical tubes: 0.64 ± 0.03; containers: 0.67 ± 0.03). In contrast, species had no significant effect (Table 2). Wilcoxon tests further showed that, except for the control condition, subjects performed above chance levels in all the other conditions (containers: *p* = 0.002; vertical tubes: *p* < 0.001; crossed tubes: *p* = 0.011).

## Discussion

In this study, we aimed to assess how different naïve physical cognitive skills were distributed across captive groups of different ungulate species, with a special focus on object permanence, short-term spatial memory, causality, and understanding of object properties and gravity. We showed that study subjects were able to understand that objects continue to exist even when they are out of sight, remember the location of objects after delays of up to 60 s (short-term spatial memory), and infer the location of the food from the presence or lack of sound produced when shaking containers. Furthermore, we showed that study subjects had some understanding of object properties and gravity, being able to locate food behind one of the two occluders based on their shape and inclination, and searching for falling food in the correct location. Our study also showed little variation across species, except for the object property task, where the performance by goats and llamas was better than the one by guanacos, zebras, and rhinos. Importantly, study subjects performed at chance levels in all the control conditions, showing that they did not rely on olfactory cues or other inadvertent cues provided by the experimenter to solve these tasks.

As predicted (Prediction 1), individuals across species performed above chance in the object permanence condition (see Fig. 4), which is a widespread ability in vertebrates ^7^ and is thought to be part of individuals’ core knowledge^5,51,54^. This ability seems indeed crucial not only to locate objects (e.g., food or predators) that remain temporarily out of view but also to locate other group members that may not always be visible in the group^32^. In line with this, study subjects were also successful in the short-term spatial memory task, as they could remember the spatial position of a hidden object in one of the two containers after delays of up to 60 s (see Fig. 4). This is in line with other studies in ungulates, showing that horses are able to remember the position of hidden food for at least 20 s^41^, while giraffes can remember the correct position for up to 30 s^33^. In the future, it would be especially interesting to assess subjects’ performance after longer delays, as many species have exceptionally long-term memory that can span from days to months^102^. Large herbivores, in particular, like zebras, also rely on long-term memory skills during migrations^103,104^, and might have evolved specialized memory systems to store biologically relevant information for long periods^102^. In this study, however, we did not evidence any differences across species in their short-term spatial memory skills, in contrast to our predictions (Predictions 2a, 2b, 2c).

In the causality task, study subjects could infer the position of food, not only based on the presence of sound when shaking the baited container but also on the absence of acoustic cues when shaking the empty container (i.e., inference by exclusion; see Fig. 4). These results suggest that animals did not simply rely on stimulus enhancement to make their choice (i.e., choosing the box that was more salient because it was shaken). Our results are in line with performance in great apes^48^, but see^105^, tufted capuchin monkeys (*Cebus apella*;^106^), and grey parrots (*Psittacus erithacus*;^107^), who were also able to locate food when the experimenter shook the non-baited box. However, other species have failed to show inference by exclusion in the auditory modality, including olive baboons (*Papio hamadryas anubis*;^108^) and dogs (*C. familiaris*;^93^). The fact that ungulates might perform better than some primate species in the same task may be surprising. There are at least two possible explanations for these results. First, causal understanding might not only be important during foraging or to predict conspecifics’ behaviour in specific situations^46^ but also to predict the behavior of heterospecific competitors, especially predators^109^. Ungulates, as compared to many other species, experience an extremely high predation pressure^110–113^, and might as a taxon have evolved a series of cognitive skills that are especially useful to deal with this challenge. Second, although we administered very few trials to avoid learning effects during our study, it is still possible that study subjects solved the task thanks to the knowledge they had gained through experience on containers and acoustic cues, rather than due to their inference skills. In particular, although none of the study subjects received any training or test providing relevant information to solve the causality task, it is true that the zoo keepers in Leipzig may bring food to ungulates in plastic buckets, which may produce sound when keepers walk through the enclosures with full buckets, but not when the buckets are empty. Regular exposure to these feeding practices might have provided our study subjects with experience useful to successfully solve the causality task, including the harder condition implying inference by exclusion. This seems to be indeed the case, as we did not find any differences across species suggestive of evolutionary pressures linked to an increase in inference skills (in contrast to Predictions 2a, 2b, 2c). Nonetheless, even if our study subjects had learned to associate the presence/absence of food with the presence/absence of sound during their feeding routine at the zoo, it is remarkable that they promptly extended this association to the novel experimental context we used. In the future, it would be important to include other study groups that are not exposed to similar feeding practices, and further test our study subjects in other tasks requiring inference skills but using a different experimental setting.

In the object property task, study subjects were able to locate hidden objects based on the size and inclination of two different occluders, suggesting that they understood that objects occupy space and have specific dimensions (see Fig. 6). In humans, this is thought to be an innate ability belonging to our core knowledge^5,51,54–56,58^, and some authors have also suggested this for other species, as it may have crucial adaptive value (e.g., to allow predictions about where possible predators might hide^22,60^. In our study, this task was the only one suggesting differences between species. In particular, goats and llamas performed better than guanacos, zebras and rhinos (see Fig. 5), suggesting that, largely in line with our Prediction 2c, domesticated species might perform better than non-domesticated ones in some tasks. Through domestication, there has been a selection for skills and traits that facilitate interaction with humans and exploration, so that domesticated species might be more interested in anthropogenic objects and more likely to interact with them^22,50,77,93^. Therefore, although domestication might not be directly linked to the evolution of higher cognitive skills in the physical domain, as foraging challenges decreased during the domestication process, it might be linked to an increase in motivation and interest in human artifacts, which increase individuals’ chances of acquiring relevant information about object properties during their lifetime^29,39,50,77^. Indeed, our results are in line with other studies on ungulates, suggesting that domesticated species like horses^61^ and pigs^50^ understand basic object properties. The fact that neither dietary breadth nor fission–fusion dynamics explained performance in any of our tasks might not necessarily mean that these factors are not linked to the enhancement of specific cognitive skills. For instance, it is possible that high fission–fusion dynamics pose social challenges and are therefore only linked to an increase of certain skills in the social domain, which cannot be detected when using physical stimuli, as in our study. This explanation, however, is unlikely, as shown by other comparative studies that have found a clear link between fission–fusion dynamics and the increase of specific cognitive skills also in the physical domain^35,74,114^. Moreover, even though cognitive skills are likely partially modular and can independently emerge in response to specific social and ecological domains, it is likely that, once they have emerged, these skills are used to deal both with objects and social partners, as this is more effective^115^. Furthermore, it is interesting to note that, as for the other tasks, performance did not vary across species depending on the condition (i.e. species had an effect on performance, but not the interaction of species and condition). These results are unlikely to depend on the low power we had in our analyses: in the model for the object permanence task, for instance, the interaction between species and condition did not reach significance, but had the rhinos failed in two further trials of the shake empty condition (i.e. with a further 6% decrease in their performance in this condition), the interaction would have become significant (*p* = 0.037), suggesting that low power was not an issue in this study.

**Figure 7:**
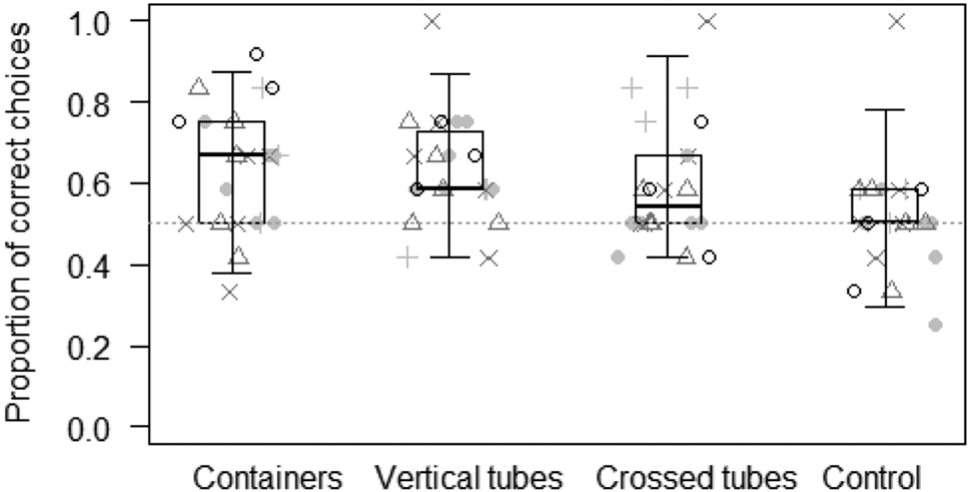
Proportion of correct choices in each condition of the object gravity task. Light grey dots represent average values for each individual goat, light grey pluses for each guanaco, dark grey triangles for each llama, dark grey crosses for each rhino, and black circles for each zebra. Thick lines represent the median of the individual values for each condition, the horizontal ends of the box represent the 25% and 75% quartiles, and the ends of the whiskers represent the 2.5% and 97.5% quartiles. The grey dotted line represents chance level.

In the object gravity task, study subjects were able to follow the trajectory of falling objects across different conditions (see Fig. 7). Although the performance was above chance in all the conditions, there was a slight decrease in performance from the easier to the harder conditions (containers: 67% of correct choices; vertical tubes: 64%; crossed tubes: 60%), suggesting that in the crossed tubes condition subjects had difficulties overcoming their gravity bias to correctly locate the food, as also found in other species (e.g., great apes:^66^; dogs:^68^; rhesus (*M. mulatta*) and stump-tailed macaques^71^).

Finally, it is important to note that, in this study, animals were rewarded for making a correct choice in the experimental trials. This procedure is extremely common in studies of animal cognition, as rewarding individuals for their correct choice is usually necessary not only to motivate animals to make choices in experimental set-ups, but also to test their understanding of different conditions. However, rewarding individuals for their correct choices might also lead them to master conditions through learning processes. In this study, in order to reduce the risk of learning effects and assess their impact on individuals’ performance, we took two different measures. First, we minimized individuals’ chances of learning by running a limited number of trials for each experimental condition (i.e., 12), as often done in literature. Second, we used a statistical approach that specifically allowed us to test for learning effects (i.e. for the effect of trial number) on individuals’ performance. In our case, all the models clearly showed that individuals’ performance did not increase through time, and that success in this study could not be explained by learning processes.

Overall, our study subjects performed quite well in the tasks and conditions we administered, showing a variety of cognitive skills in the physical domain. These results are in line with several studies that show evidence of different cognitive skills across ungulate species, including horses, goats, pigs, cows, and several non-domesticated species. Across species, we found relatively little variation, which might however also depend on the small sample sizes we used. In the future, it will be especially important to include more subjects and species to confirm our preliminary findings, test other hypotheses of cognitive evolution, and better understand the limits and generalizability of these hypotheses. In this regard, ungulates appear to be a highly valuable model for the study of comparative cognition, given that the large variety of socio-ecological characteristics (e.g. dietary breadth, domestication, social structure) shown by these species can allow to reliable contrast different evolutionary hypotheses and detect cases of convergent evolution.

## Data Availability

Data are made available at figshare, 10.6084/m9.figshare.25101848.
